# GOI-YOLOv8 Grouping Offset and Isolated GiraffeDet Low-Light Target Detection

**DOI:** 10.3390/s24175787

**Published:** 2024-09-05

**Authors:** Mengqing Mei, Ziyu Zhou, Wei Liu, Zhiwei Ye

**Affiliations:** School of Computer Science, Hubei University of Technology, Wuhan 430068, China; meimengqing@hbut.edu.cn (M.M.); 102211184@hbut.edu.cn (Z.Z.); 20071014@hbut.edu.cn (W.L.)

**Keywords:** low-light conditions, grouping offset, YOLOv8, Isolated GiraffeDet

## Abstract

In the realm of computer vision, object detection holds significant importance and has demonstrated commendable performance across various scenarios. However, it typically requires favorable visibility conditions within the scene. Therefore, it is imperative to explore methodologies for conducting object detection under low-visibility circumstances. With its balanced combination of speed and accuracy, the state-of-the-art YOLOv8 framework has been recognized as one of the top algorithms for object detection, demonstrating outstanding performance results across a range of standard datasets. Nonetheless, current YOLO-series detection algorithms still face a significant challenge in detecting objects under low-light conditions. This is primarily due to the significant degradation in performance when detectors trained on illuminated data are applied to low-light datasets with limited visibility. To tackle this problem, we suggest a new model named Grouping Offset and Isolated GiraffeDet Target Detection-YOLO based on the YOLOv8 architecture. The proposed model demonstrates exceptional performance under low-light conditions. We employ the repGFPN feature pyramid network in the design of the feature fusion layer neck to enhance hierarchical fusion and deepen the integration of low-light information. Furthermore, we refine the repGFPN feature fusion layer by introducing a sampling map offset to address its limitations in terms of weight and efficiency, thereby better adapting it to real-time applications in low-light environments and emphasizing the potential features of such scenes. Additionally, we utilize group convolution to isolate interference information from detected object edges, resulting in improved detection performance and model efficiency. Experimental results demonstrate that our GOI-YOLO reduces the parameter count by 11% compared to YOLOv8 while decreasing computational requirements by 28%. This optimization significantly enhances real-time performance while achieving a competitive increase of 2.1% in Map50 and 0.6% in Map95 on the ExDark dataset.

## 1. Introduction

In the past few years, there has been notable advancement in advanced object detection systems [1,2,3,4,5,6,7], and various benchmark datasets have achieved satisfactory results under real-time constraints. Among them, the YOLO [8,9,10,11] series represents a single-stage end-to-end detector with an efficient network structure [12,13,14] and advanced training stage [15], and has been widely adopted in industry. However, most existing detectors are primarily studied under normal illumination conditions, which poses challenges when encountering dark environments, insufficient lighting, and exposure time conditions, resulting in performance degradation, low visibility, color distortion, and noise [16,17]. These factors contribute to a decline in image lighting quality and hinder the accuracy of object detection. Two common methods are typically employed to address this issue.

First, in low-light image enhancement (LLIE) [18,19,20,21,22,23,24,25,26] it is challenging to restore scene details and improve visibility by mitigating adverse lighting conditions. However, existing models have several limitations: (1) the complex structure of current low-light enhancement models often hinders real-time performance for detection tasks, requiring separate image enhancement before detection; (2) while low-light image enhancement can push the limits of human visual perception, the lack of multiscale features typically results in increased brightness only in specific areas, leading to loss of edge and texture information within the scene; (3) low-light image enhancement methods usually optimize network performance by improving loss functions, however, this approach forces the network to adopt a uniform learning method for all pixels, thereby sacrificing detailed information learning; (4) existing low-light image enhancement techniques often depend on paired training data that include images captured in low-light conditions along with images taken in bright lighting [27,28].

Second, in low-light target detection, the detector is typically trained on well-lit images and undergoes slight adjustments when applied to low-light images [29,30,31,32]. The network structure of the detector is enhanced to improve its ability to detect potential information under low-light conditions. The benefits of utilizing this method are that: (1) it allows for end-to-end training and exhibits strong real-time performance; (2) the design architecture preserves the inherent scene information without compromising its integrity; (3) better extraction of potential low-light information from the scene allows the network architecture to achieve effective detection while retaining detailed scene information; (4) the detector can be directly utilized in low-light scenarios without requiring paired low-light and bright images for training [33,34].

Combined with the aforementioned methodologies, this paper considers a real-time performance-oriented low-light target detection method. It is imperative for the detector architecture to effectively learn and integrate fusion information between high-level spatial features and low-level spatial features, thereby making the feature fusion layer of the detector a crucial component within the overall framework. Use of the Feature Pyramid Network (FPN) [35] has been validated as an effective approach for fusing multiscale features in detectors. However, there remains an unresolved challenge regarding the fusion of potential information present in low-light images. In this study, we refer to DAMO-YOLO’s RepGFPN neck fusion structure [36] and enhance its feature fusion capability by incorporating additional layers [37,38,39]. Our objective is to augment the feature fusion depth of the model’s neck structure beyond that of FPN-PAN, enabling multilevel fusion and better utilization of potential information within low-light datasets.

However, the RepGFPN neck structure eliminates the excessive upsampling structure of the original GFPN module, resulting in a lack of information sampling ability for low-light scene detection targets. Therefore, The RepGFPN architecture lacks a well-designed single-input upsampling module to mitigate the loss of information features caused by the insufficient number of upsampling modules in its design. Consequently, we require a simple, fast, cost-effective, and versatile upsampler that can also enhance the performance of the upsampling module for potential information extraction in low-light scenes. Drawing inspiration from DySample [40], we assume that the input feature values possess continuous characteristics similar to bilinear interpolation, and propose a method to resample this continuous mapping using content-aware sampling points. By controlling the initial sampling positions and adjusting the offset movement ranges, we refine our new upsampling module by reorganizing the process into several independent groups. Through control over offsets and grouping operations, we isolate issues related to detecting target edge information confusion in low-light scenes and address deficiencies within GFPN’s structure. Experimental results demonstrate that integrating DySample with RepGFPN effectively compensates for RepGFPN’s shortcomings regarding potential information sampling in low-light scenes due to an excess number of missing upsampling modules.

**Figure 1:**
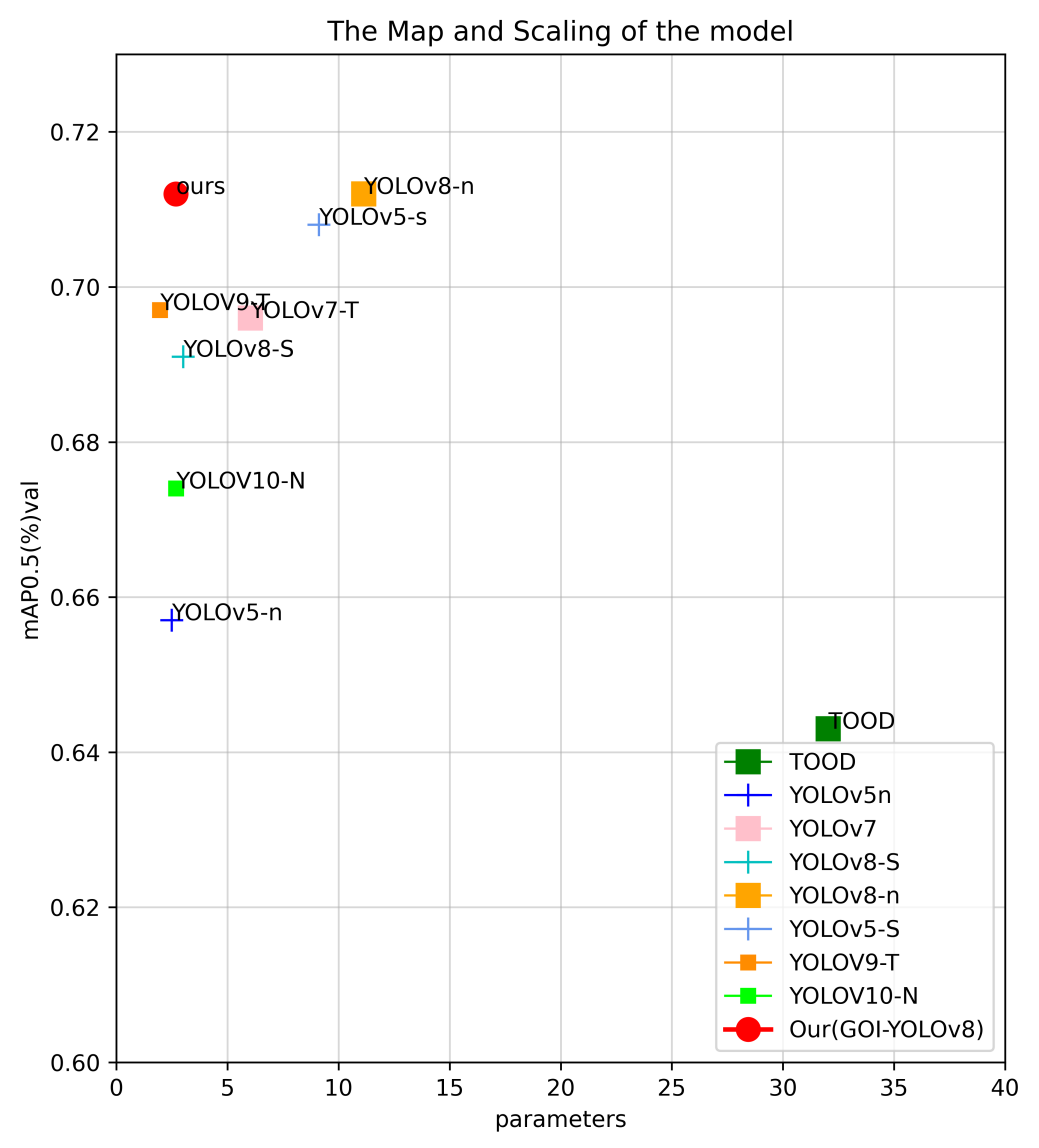
Comparison of parameter accuracy among leading object detectors.

After improving the neck feature fusion layer of the network structure, we conducted a series of experiments to improve the detection head with the aim of achieving a balance between real-time performance and accuracy. We considered that the key to low-light scene detection is to isolate the confusion of scene edge detection information and the group isolation effect of group convolution. Finally, we replaced the convolution in the detection head module with group convolution to isolate the interference information of the detected object edge and improve the detection performance. In addition to making up for the loss of real-time performance due to the modification of the neck, this approach achieves competitive results in terms of accuracy. The comparison of the parameter accuracy of the main object detectors is shown in Figure 1.

Our contribution can be summarized as follows:

- The original neck part of YOLOv8 is reconstructed based on the repGFPN feature fusion network to achieve multilevel fusion in order to better use the potential information in the low-light dataset for feature fusion.
- The GFPN network architecture is enhanced by incorporating the concept of Dysample offset, aiming to address the limitations of the original GFPN architecture and effectively extract deep information from low-light scenes as well as to capture potential information within the low-light dataset.
- Group convolution is employed as a substitute for conventional convolution in the detection head, effectively mitigating the blurring of edge information caused by low light conditions. By isolating interference information related to object edges, this approach enhances the compatibility between the model’s detection head and the improved network architecture, thereby contributing to a better balance between real-time performance and model accuracy.
- The proposed method demonstrates superior real-time performance and accuracy compared to the baseline model, as evidenced by numerous experiments.

## 2. Related Work

### 2.1. Object Detection

Mainstream object detection methods can generally be categorized into either one- or two-step detectors and anchor-free models.

Among these, SSD and YOLO [41] belong to the class of one-stage detectors. As one-stage detectors, they are designed to directly predict the object bounding box and class label, then immediately output the location and category information of the target. Because of their fast speed and less need for calculation, one-stage detectors usually work in scenes with high real-time requirements.

On the other hand, R-CNN, R-FCN [42], and other similar models are classified as two-stage detectors. These detectors first generate region proposals, then perform classification and bounding box regression to refine these proposals. Due to their two-stage detection process, these detectors typically require more computational resources and time. In comparison to one-stage detectors, two-stage detectors exhibit significant limitations in specific application scenarios and requirements.

Therefore, to ensure the broad applicability and real-time requirements of the detector, in this study we have opted to investigate a one-stage detector.

### 2.2. Low-Light Image Enhancement

Due to the emergence of deep learning, there has been widespread investigation into low-light image enhancement. There are two primary methods for enhancing low-light images: one involves utilizing reflectance as the enhanced image, while the other entails reconstructing the enhanced image through illumination adjustment.

The task of low-light enhancement detection aims to improve the visual perception of humans by restoring image details and correcting color distortion, thereby generating illuminated images for advanced visual tasks such as object detection. This significantly impacts the real-time performance of detectors such as Kind, zero-DCE, MBLLEN, and IAT.

### 2.3. Low-Light Object Detection

Our primary focus lies in enhancing object detection performance through network structure adjustments or the utilization of specialized neural networks, particularly when dealing with low-light images. The current research trend involves developing end-to-end models for low-light detection and applying object detectors in low-light scenes. To achieve an end-to-end system for detecting low-light scenes, this approach directly extracts potential features from the original image to enhance both detection performance and efficiency. Thus, in this paper we adopt this method and research direction. Compared to the complexity and lack of real-time performance associated with image enhancement techniques, the end-to-end low-light target detection system holds greater application value and significance.

## 3. Method

Detection performance is adversely affected by weak light interference, resulting in diminished visibility in low-light images. To harness the potential information in these images, we employ repGFPN for various levels of network feature fusion and integrate Dysample upsampling to capture scene-specific details. Additionally, a novel group convolution detection head is utilized to mitigate interference from object edges. This approach effectively eliminates blurred edge information under low-light conditions while maintaining a balance between real-time performance and model accuracy.

### 3.1. GOI-YOLO Model

In this section, we present the details of our enhanced GOI-YOLO model, which has been trained on the Exdark dataset [43] with a focus on thirteen specific classes. Our model incorporates several enhancements to achieve real-time performance, high accuracy, and improved generalization.

First, we employ the repGFPN structured feature fusion network to reconstruct the original neck component of YOLOv8. This enhancement enables the feature fusion layer to effectively improve the fusion effect by expanding its hierarchical levels, allowing the model to better fuse the high-level spatial information and low-level spatial information, which are equally important to the feature fusion layer in low-light scenes.

Second, we enhance the upsampling module in repGFPN to mitigate the loss of deep neck information features resulting from excessive removal of upsampling modules, thereby maximizing the acquisition of profound semantic information from low-light data.

**Figure 2:**
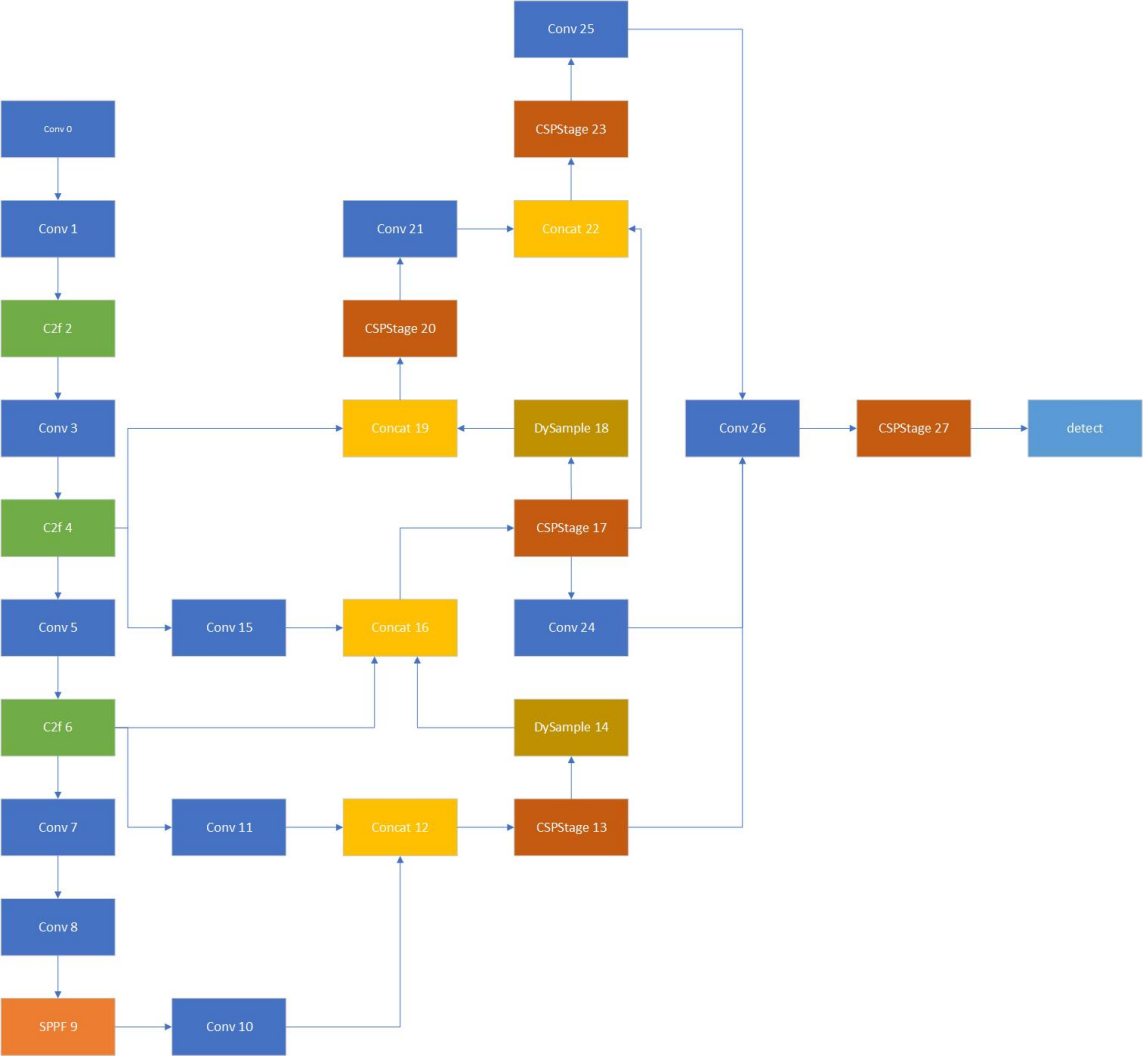
Overview of GOI-YOLO. The architecture of GOI-YOLO is based on YOLOv8, and combines the repGFPN architecture based on GFPN with Efficient Aggregation Network (ELAN) and a reparameterized CSP, DySample module, and new group convolution Detect module. The Conv, C2f, SPPF, and Concat modules already exist in the original YOLOv8 architecture.

Finally, group convolution is employed in our approach as a substitute for convolution in the detection head, achieving a more balanced trade-off between real-time performance and model accuracy. By isolating edge information from low-light scenes, our method becomes more competitive compared to the baseline model. The specific framework is illustrated in Figure 2.

### 3.2. Enhanced GFPN

FPN was initially proposed to tackle the problem of integrating hierarchical features in convolutional neural networks, and it has been empirically demonstrated that globally enhancing components effectively enhance the capability of deep learning models for object detection tasks. However, the unidirectional top-to-bottom information flow restricts the feature fusion ability of FPN.

FPANet is used to strengthen features and encourage information reuse, thereby improving the representation ability of the feature pyramid. Diverging from conventional FPN, an additional bottom-up pathway is incorporated to aggregate shallow feature maps (with low resolution but weak semantic information) and deep feature maps (with high resolution and rich semantic information) within the image. Information transmission occurs along a specific route to convey low-level image features, further strengthening the expressive capability of multi-scale features and resulting in PANet’s superior performance on detection tasks; however, this comes at higher computational cost.

The neck features of YOLOv5 and YOLOv8 are fused using FPN and PANet. The distinction between v5 and v8 lies in the latter’s replacement of the c3 module with the c2f module during the upsampling stage. Compared to FPN, PANet can accurately preserve spatial information by fusing feature maps from bottom to top. However, the combination of FPN and PANet only supports top-down and bottom-up feature fusion within the network’s fusion structure.

The design of BiFPN incorporates a higher number of connections between different layers and skip connections to enhance feature fusion. However, it is important to note that simple stacking of BiFPN may not be optimal, as excessively deep stacking can potentially lead to gradient disappearance.

GFPN utilizes dense connections and the QUEEN-FUSION structure to generate enhanced fusion features, employing a series operation instead of a summation for feature fusion to minimize information loss. However, the real-time performance of the GFPN-based model is inferior to that of the FPN-PANet model due to several underlying factors:

(1) The performance of QUEEN-FUSION fails to meet real-time detection model requirements.
(2) The efficiency of convolution-based cross-scale feature fusion is suboptimal.

**Figure 3:**
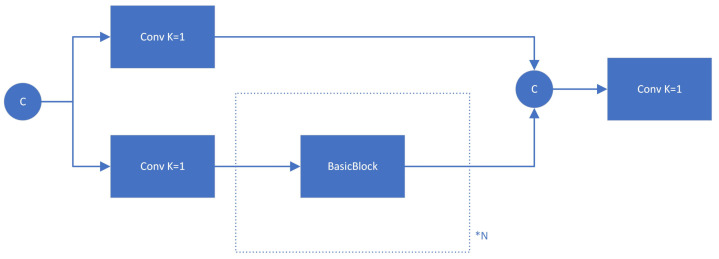
CSP module.

Based on the above defects, and inspired by DAMO-YOLO, we introduce the following:

(1) The GFPN model enhances feature interaction through QUEEN-FUSION while also introducing multiple additional upsampling operators. By eliminating a portion of the upsampling operations, the computational burden of the model is reduced.
(2) CSPNet [44] is utilized to replace the initial feature fusion based on a 3 × 3 convolution. We employ the cross-stage Partially Dense Network (CSP) in conjunction with GFPN to enhance CSPNet and optimize the neck structure, addressing the issue of low efficiency in cross-scale feature fusion based on convolution within the original architecture. The CSP module is illustrated in Figure 3.

The redundant upsampling operation is eliminated in GFPN, and CSPNet replaces the original 3 × 3 convolution in GFPN. In YOLOv8, the network structure’s neck feature fusion layer is redesigned by replacing C2f and combining it with Conv. By adding layers, the effect of feature fusion is enhanced to meet the requirements of real-time detection models while simultaneously sharing dense information at various spatial scales and non-adjacent latent semantic levels. This allows for capturing potential information in the fused image, enabling simultaneous processing of high-level and low-level spatial information that is equally crucial to the neck region, thereby maximizing potential information extraction in low-light scenes.

### 3.3. Upsampling Based on the Idea of Offset

The repGFPN structure eliminates most of the upsampling modules in GFPN, resulting in an insufficient number of upsampling modules in the improved neck feature fusion module. This fails to meet the requirements of simplicity, speed, and efficiency, while also compromising the ability to extract features through upsampling. In the offset-based upsampling process, after interpolating input features using bilinear interpolation, content-aware sampling points are generated to resample continuous images. By controlling offsets and grouping operations, this approach effectively addresses the issue of target edge information confusion detection in low-light scenes and compensates for the limited sampling performance of GFPN’s original upsampling structure under such conditions.

The essence of the upsampling module is point sampling. Given a feature map *X* of size and a sampling set *S* of size, where 2 in the first dimension denotes the x and y coordinates, the grid sample function uses the positions in *S* to resample the hypothetical bilinear-interpolated *X* into of size. This process is defined by

(1) $$X^{\prime }=grid\_sample\left(X,S\right)\;.$$

#### 3.3.1. Offset Upsampling Implementation

**Figure 4:**
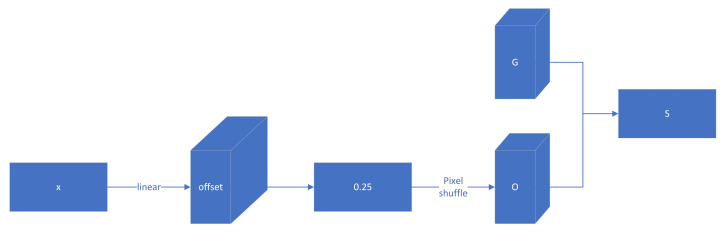
Dysample module.

Given an upsampling scale factor s and a feature map *X* of size, a linear layer with input and output channel numbers C is used to generate the offset *O* of size, which is then reshaped by Pixel Shuffling [36]. Then, the sampling set *S* is the sum of the offset *O* and the original sampling grid *G*, i.e.,

The offset process is shown in Figure 4.

(2) O=linear(X)

(3) S=G+O

#### 3.3.2. Offset Scope

Due to the existence of normalization layers, the values of one certain output feature are typically in the range of [1, 1], centered at 0. Therefore, the walking scope of the local sampling positions could overlap significantly. This overlap could easily influence the prediction near boundaries, and such errors would propagate stage by stage and cause output artifacts. To alleviate this, we follow the work of [41] by multiplying the offset by a weight coefficient; in this paper, we rewrite (2) as follows:

(4) O=β×linear(X).

The performance of Map50 is enhanced to 0.712 when the range factor is set at 0.25. Experimental results demonstrate that this coefficient precisely satisfies the theoretical marginal condition between overlap and non-overlap, referred to as the ‘static scope coefficient’. Consequently, it locally restricts the walking range of sampling positions, as illustrated in Table 1.

#### 3.3.3. Grouping

The same sampling set for features is shared among each group. Considering the potential confusion caused by the association between data features and surrounding environment feature information in low-light conditions, group upsampling can effectively isolate the information exchange among different groups. To achieve this, we divide the feature map into g groups along the channel dimension and generate corresponding offsets for each group. Through experimental verification, as illustrated in the Table 2, we have determined that g should be set to 4.

Our experiments demonstrate that static offset upsampling using DySample not only exhibits superior performance but also incurs the lowest costs in terms of inference latency, training memory, training time, GFLOPs, and parameter count when compared to previous upsamplers. Concerning inference time, DySample’s backpropagation is notably swift due to its utilization of highly optimized built-in PyTorch functions; however, the additional training time incurred is negligible.

The input feature map is divided into g groups based on the channel in group convolution, followed by regular convolution performed on each group. This approach reduces both computation and parameter requirements while maintaining the same input and output size. When the input and output feature maps are associated, group convolution outperforms regular convolution. For instance, under low-light conditions during the mixing stage of image processing, grouped convolution can be employed to assign zero weights to irrelevant input channels directly, thereby eliminating interference from blurred edge information.

We have opted to replace the original convolution with group convolution to eliminate the computational burden of neck feature fusion and facilitate isolated information exchange among different groups under low light conditions, thereby enhancing detection accuracy to its fullest potential. We conducted numerous replacement experiments after abandoning the initial convolution. The specific replacements are detailed in Table 3.

### 3.4. Improved Detection Head

The real-time performance and accuracy of the model was not well balanced, despite using GFPN to fuse ELAN and reparameterizing the CSP neck structure modification. To ensure a balanced model, we made modifications to the detection head, aiming to improve both FPS and accuracy while decreasing the number of parameters and computational complexity.

Considering the confusion caused by the correlation between the data feature information and surrounding environment feature information in low-light data, as well as the results previously obtained by grouping upsampling, we use group convolution in the detection head to isolate the information exchange between different groups.

**Figure 5:**
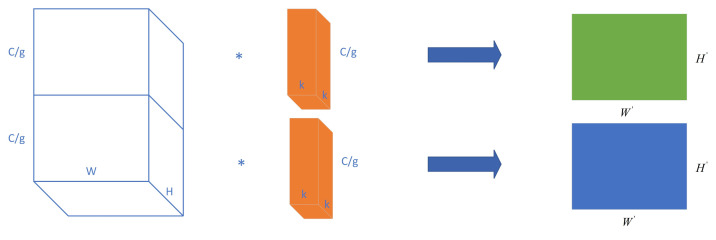
Group convolution. * is the convolution operation.

<1> We use 1× 1Conv + 3 × 3Conv to replace the original convolution, hoping to achieve better results and accuracy through the combination of convolutions of different sizes.
<2> We use Efficient Multi-Scale-Conv (EMSC), a multiscale convolutional network architecture that can apply multiscale convolutional operations on input images at different scales, to capture different features in the image and obtain richer feature representations by cascading operations on feature maps at different scales.
<3> We use EMSCP, an improved version of EMSC which introduces a position-aware module based on EMSC, to improve the model’s capacity to recognize unknown information.
<4> We employ the SCNet self-calibrated convolution module, which utilizes a low-dimensional embedding transformed by a single convolution kernel to calibrate the convolution transform of the other part’s kernel. This approach effectively enhances the receptive field at each spatial location.
<5> We use group convolution in the Figure 5, which is expected to block out noisy information about the edges of the detected objects.

The experimental results presented in this paper confirm the effectiveness of the aforementioned method. The choice of group convolution as a replacement for the original convolution effectively mitigates edge confusion in low-light scenes for detection targets. Moreover, employing group convolution not only enhances accuracy but also significantly reduces the computational burden associated with neck feature fusion.

## 4. Experiments

Our model was trained for 400 cycles using the SGD optimizer with an initial learning rate of 0.01 and batch size of 32. We ran our model on a single RTX 4060 TI GPU.

### 4.1. Dataset

We validated the effectiveness of GOI using the ExDark dataset, a low-light object detection dataset for object detection and image enhancement research. The ExDark dataset is partially derived from various public datasets. Unlike common object datasets, this dataset consists entirely of low-light images captured in visible light, with image and object level annotations of up to twelve classes as well as up to ten distinct types of low-light conditions. We divided ExDark into train, van, and test sets in a ratio of 8:1:1; the final experimental accuracy and other data were verified on the test set.

### 4.2. Evaluation

The evaluation criteria were the most commonly used and authoritative indicators in object detection: the number of model parameters, number of model calculations, model FPS, and accuracy index (map50).

### 4.3. Experimental Results

To prove the effectiveness of GOI-YOLO model, we conducted an experimental evaluation on the ExDark dataset. We compared GOI-YOLO with other object detection models. Our GOI-YOLO model scores 2.1/0.1% higher than the baseline YOLOv8-S/N model in terms of map50. Compared with YOLOv7-T, YOLOv5-S/N, YOLOV9-T, YOLOV10-N, and TOOD, it achieves the best effect by 1.6%, 5.5/0.4%, 0.5%, 3.8%, and 6.9%, respectively. Our model has the highest FPS and lowest number of parameters, and its number of parameters is 9% lower than that of the baseline YOLOv8-S model. Our model reduces the number of parameters by 75% and has on the slightly higher accuracy in terms of map50 compared to YOLOv8-N. The data in Table 4 show that our model is the most suitable for the task of object detection in low-light scenes.

**Figure 6:**
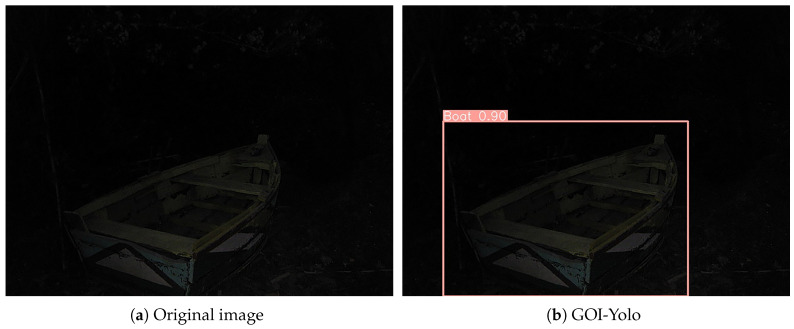

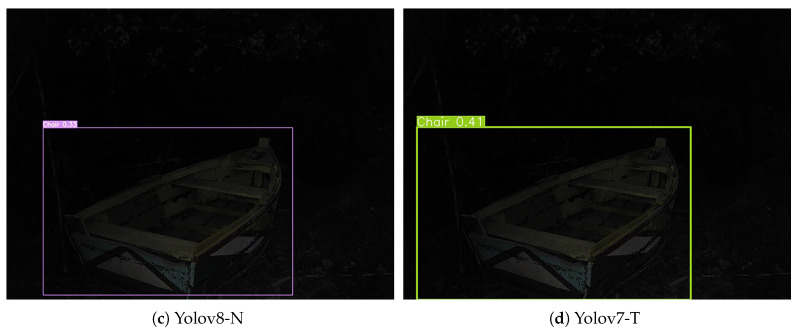
Detection results for the proposed GOI-YOLO model and other YOLO-series detectors.

The detection results of the different detectors are visualized in Figure 6. It can be observed that the proposed GOI-YOLO model effectively isolates the influence of edge information in low-light scenes, resulting in more accurate prediction boxes and precise capture of potential object information in low-light images.

The primary disadvantage of mainstream detection models is that they are typically evaluated under well-lit conditions, while specialized datasets for low-light environments are scarce. Currently, the accuracy of these models is constrained by the limited availability of low-light datasets, which may result in insufficient training data. We believe an adequate supply of relevant datasets could allow the model proposed in this paper to better capture target features under low-light conditions, thereby improving its accuracy.

### 4.4. Ablation Study

The advantages of incorporating each method into the model and their respective impacts on model performance were evaluated through a series of ablation experiments. As can be seen in Table 5, after using repGFPN, although the Map of our model increases from 69.1% to 70.4%, indicating that it is effective in improving feature fusion by adding layers, it also leads to a 7.6% increase in parameters. After adopting DySample, the Map50-95 is increased from 43.8 to 44.1%, which indicates that the sampling offset method of DySample can obtain more deep information in low-light scenes. After using Gconv, the Map50 is increased from 70.4% to 71.2%, indicating that group convolution can separate the interference information of the detected object edge to improve the detection performance, while the number of parameters decreases from 3.26 m to 2.67 m, leading to comprehensive improvement in various indicators.

## 5. Conclusions

The application of low-light detection has had a significant impact on various practical scenarios, including autonomous driving, selfies, and nighttime rescue missions. Our model is specifically designed to cater to low-light scenes with limited illumination quality. To achieve real-time target detection in such scenes for deployment on edge devices, we have developed the GOI-YOLO dark target detection framework. GOI-YOLO is trained end-to-end, enabling the network to recover details and capture potential information. Additionally, the RepGFPN fusion network is employed to enhance feature fusion levels. We utilize the Dysample sampling offset method to extract deep information from low-light scenes, and employ group convolution to isolate interference information at the edges of detected objects for improved performance without compromising real-time capabilities. Experimental evaluations were conducted using the ExDark dataset, demonstrating that GOI-YOLO outperforms mainstream detectors by further enhancing performance while maintaining its lightweight nature and effectively detecting targets in low-light environments. During our experiments, we observed that the model’s accuracy may decline due to the loss of essential information in low-light conditions. It is important to distinguish between low-light conditions and a complete absence of lighting, as accurate detection of real-world scenes requires the presence of some lighting and light source information.

## Figures and Tables

**Table 1:** Experiments on static scope coefficient.

β	Map50
0.1	0.694
0.2	0.69
0.25	0.712
0.3	0.692
0.5	0.702

**Table 2:** Grouping experiments on grouping upsampling.

g	Map50
1	0.692
4	0.712
8	0.686

**Table 3:** Detection head replacement experiments.

Methods	Params	GFLOPs	Map50	Map50-95
baseline	3.26 m	8.4	0.701	0.441
1× 1Conv + 3 × 3Conv	3.41 m	7.1	0.701	0.433
EMSConv	2.90 m	6.2	0.692	0.437
EMSConvP	3.17 m	6.7	0.691	0.433
SCNet	2.79 m	6.0	0.682	0.416
GConv	**2.68 m**	**5.9**	**0.712**	**0.441**

**Table 4:** Comparison with current mainstream object detection models on the ExDark dataset, showing the detection results of each model in terms of Params, GFLOPs, FPS, Map50, and MAP50-95.

Methods	Params	GFLOPs	FPS	Map50	Map50-95
YOLOV5-S	2.50 m	7.2	212	0.657	0.397
YOLOV5-N	9.12 m	24.0	158	0.708	0.436
YOLOV7-T	6.03 m	13.1	161	0.696	0.400
YOLOV8-S	3.01 m	8.2	263	0.691	0.435
YOLOV8-N	11.13 m	28.6	158	0.711	0.449
YOLOV9-T	1.97 m	7.6	227	0.697	0.441
YOLOV10-N	2.69 m	8.3	289	0.674	0.42
TOOD	32.044 m	199	69	0.643	0.444
GOI-YOLO	2.68 m	**5.9**	**290**	**0.712**	0.441

**Table 5:** Ablation experiments on the ExDark dataset.

Methods	Params	GFLOPs	FPS	Map50	Map50-95
**Baseline**	3.01 m	8.2	263	0.691	0.435
**+repGFPN**	3.26 m	8.4	277	0.704	0.438
**+DySample**	3.02 m	8.2	285	0.689	0.431
**+GConv**	2.42 m	5.8	294	0.69	0.43
**+DySample+GConv**	2.43 m	**5.7**	256	0.687	0.431
**+repGFPN+GConv**	2.67 m	5.9	192	0.701	0.436
**+repGFPN+DySample**	3.26 m	8.4	287	0.701	0.441
**+repGFPN+DySample+GConv**	2.68 m	5.9	**290**	**0.712**	**0.441**

## Data Availability

The data is from the paper Getting to Know Low-light Images with The Exclusively Dark Dataset. It is available at https://github.com/cs-chan/Exclusively-Dark-Image-Dataset.

## References

[1] Bochkovskiy A., Wang C.Y., Liao H.Y.M. (2020). Yolov4: Optimal speed and accuracy of object detection. arXiv.

[2] Ge Z., Liu S., Wang F., Li Z., Sun J. (2021). Yolox: Exceeding yolo series in 2021. arXiv.

[3] Girshick R. Fast r-cnn. Proceedings of the IEEE International Conference on Computer Vision.

[4] Liu W., Anguelov D., Erhan D., Szegedy C., Reed S., Fu C.Y., Berg A.C. (2016). Ssd: Single shot multibox detector. Proceedings of the Computer Vision–ECCV 2016: 14th European Conference.

[5] Redmon J., Divvala S., Girshick R., Farhadi A. You only look once: Unified, real-time object detection. Proceedings of the IEEE Conference on Computer Vision and Pattern Recognition.

[6] Ren S., He K., Girshick R., Sun J. (2015). Faster r-cnn: Towards real-time object detection with region proposal networks. Advances in Neural Information Processing Systems.

[7] Wang C.Y., Bochkovskiy A., Liao H.Y.M. Scaled-yolov4: Scaling cross stage partial network. Proceedings of the IEEE/CVF Conference on Computer Vision and Pattern Recognition.

[8] Li C., Guo C.L., Zhou M., Liang Z., Zhou S., Feng R., Loy C.C. (2023). Embedding fourier for ultra-high-definition low-light image enhancement. arXiv.

[9] Li C., Li L., Jiang H., Weng K., Geng Y., Li L., Ke Z., Li Q., Cheng M., Nie W. (2022). YOLOv6: A single-stage object detection framework for industrial applications. arXiv.

[10] Wang C.Y., Bochkovskiy A., Liao H.Y.M. YOLOv7: Trainable bag-of-freebies sets new state-of-the-art for real-time object detectors. Proceedings of the IEEE/CVF Conference on Computer Vision and Pattern Recognition.

[11] Chen R.C., Dewi C., Zhuang Y.C., Chen J.K. (2023). Contrast limited adaptive histogram equalization for recognizing road marking at night based on YOLO models. IEEE Access.

[12] Chen Y., Yang T., Zhang X., Meng G., Xiao X., Sun J. (2019). Detnas: Backbone search for object detection. Adv. Neural Inf. Process. Syst..

[13] Jiang Y., Tan Z., Wang J., Sun X., Lin M., Li H. (2022). Giraffedet: A heavy-neck paradigm for object detection. arXiv.

[14] Sun Z., Lin M., Sun X., Tan Z., Li H., Jin R. (2021). Mae-det: Revisiting maximum entropy principle in zero-shot nas for efficient object detection. arXiv.

[15] Tan M., Pang R., Le Q.V. Efficientdet: Scalable and efficient object detection. Proceedings of the IEEE/CVF Conference on Computer Vision and Pattern Recognition.

[16] Sen P., Das A., Sahu N. (2021). Object detection in foggy weather conditions. Proceedings of the International Conference on Intelligent Computing & Optimization.

[17] Li W., Guo X., Yuan Y. Novel Scenes & Classes: Towards Adaptive Open-set Object Detection. Proceedings of the IEEE/CVF International Conference on Computer Vision.

[18] Guo C., Li C., Guo J., Loy C.C., Hou J., Kwong S., Cong R. Zero-reference deep curve estimation for low-light image enhancement. Proceedings of the IEEE/CVF Conference on Computer Vision and Pattern Recognition.

[19] Guo X., Li Y., Ling H. (2016). LIME: Low-light image enhancement via illumination map estimation. IEEE Trans. Image Process..

[20] Cai Y., Bian H., Lin J., Wang H., Timofte R., Zhang Y. Retinexformer: One-stage retinex-based transformer for low-light image enhancement. Proceedings of the IEEE/CVF International Conference on Computer Vision.

[21] Jiang Y., Gong X., Liu D., Cheng Y., Fang C., Shen X., Yang J., Zhou P., Wang Z. (2021). Enlightengan: Deep light enhancement without paired supervision. IEEE Trans. Image Process..

[22] Jin Y., Yang W., Tan R.T. (2022). Unsupervised night image enhancement: When layer decomposition meets light-effects suppression. Proceedings of the 17th European Conference on Computer Vision.

[23] Dudhane A., Zamir S.W., Khan S., Khan F.S., Yang M.H. Burst image restoration and enhancement. Proceedings of the IEEE/cvf Conference on Computer Vision and Pattern Recognition.

[24] Wu Y., Pan C., Wang G., Yang Y., Wei J., Li C., Shen H.T. Learning semantic-aware knowledge guidance for low-light image enhancement. Proceedings of the IEEE/CVF Conference on Computer Vision and Pattern Recognition.

[25] Xu K., Yang X., Yin B., Lau R.W. Learning to restore low-light images via decomposition-and-enhancement. Proceedings of the IEEE/CVF Conference on Computer Vision and Pattern Recognition.

[26] Xu X., Wang R., Fu C.W., Jia J. Snr-aware low-light image enhancement. Proceedings of the IEEE/CVF Conference on Computer Vision and Pattern Recognition.

[27] Wei C., Wang W., Yang W., Liu J. (2018). Deep retinex decomposition for low-light enhancement. arXiv.

[28] Wu W., Weng J., Zhang P., Wang X., Yang W., Jiang J. Uretinex-net: Retinex-based deep unfolding network for low-light image enhancement. Proceedings of the IEEE/CVF Conference on Computer Vision and Pattern Recognition.

[29] Sasagawa Y., Nagahara H. (2020). Yolo in the dark-domain adaptation method for merging multiple models. Proceedings of the Computer Vision–ECCV 2020: 16th European Conference.

[30] Wang W., Yang W., Liu J. Hla-face: Joint high-low adaptation for low light face detection. Proceedings of the IEEE/CVF Conference on Computer Vision and Pattern Recognition.

[31] Wang W., Wang X., Yang W., Liu J. (2022). Unsupervised face detection in the dark. IEEE Trans. Pattern Anal. Mach. Intell..

[32] Wang W., Xu Z., Huang H., Liu J. Self-aligned concave curve: Illumination enhancement for unsupervised adaptation. Proceedings of the 30th ACM International Conference on Multimedia.

[33] Lengyel A., Garg S., Milford M., van Gemert J.C. Zero-shot day-night domain adaptation with a physics prior. Proceedings of the IEEE/CVF International Conference on Computer Vision.

[34] Luo R., Wang W., Yang W., Liu J. Similarity min-max: Zero-shot day-night domain adaptation. Proceedings of the IEEE/CVF International Conference on Computer Vision.

[35] Ghiasi G., Lin T.Y., Le Q.V. Nas-fpn: Learning scalable feature pyramid architecture for object detection. Proceedings of the IEEE/CVF Conference on Computer Vision and Pattern Recognition.

[36] Xu X., Jiang Y., Chen W., Huang Y., Zhang Y., Sun X. (2022). Damo-yolo: A report on real-time object detection design. arXiv.

[37] Sun L., Li C., Ding X., Huang Y., Chen Z., Wang G., Yu Y., Paisley J. (2022). Few-shot medical image segmentation using a global correlation network with discriminative embedding. Comput. Biol. Med..

[38] Li C., Ma W., Sun L., Ding X., Huang Y., Wang G., Yu Y. (2022). Hierarchical deep network with uncertainty-aware semi-supervised learning for vessel segmentation. Neural Comput. Appl..

[39] Li W., Chen Z., Li B., Zhang D., Yuan Y. (2021). Htd: Heterogeneous task decoupling for two-stage object detection. IEEE Trans. Image Process..

[40] Liu W., Lu H., Fu H., Cao Z. Learning to Upsample by Learning to Sample. Proceedings of the IEEE/CVF International Conference on Computer Vision.

[41] Tian Z., Chu X., Wang X., Wei X., Shen C. (2022). Fully convolutional one-stage 3d object detection on lidar range images. Adv. Neural Inf. Process. Syst..

[42] Dai J., Li Y., He K., Sun J. (2016). R-fcn: Object detection via region-based fully convolutional networks. Adv. Neural Inf. Process. Syst..

[43] Loh Y.P., Chan C.S. (2019). Getting to know low-light images with the exclusively dark dataset. Comput. Vis. Image Underst..

[44] Wang C.Y., Liao H.Y.M., Wu Y.H., Chen P.Y., Hsieh J.W., Yeh I.H. CSPNet: A new backbone that can enhance learning capability of CNN. Proceedings of the IEEE/CVF Conference on Computer Vision and Pattern Recognition Workshops.

